# Big Data Sharing: A Crucial Democratic Issue for Genomic Medicine

**DOI:** 10.3389/fpubh.2018.00334

**Published:** 2018-11-28

**Authors:** Benjamin Derbez

**Affiliations:** Université de Bretagne Occidentale, Brest, France

**Keywords:** big data, genomics, BRCA, oncogenetics, database

## Introduction

Big data are often viewed as responsible for major upheavals in many aspects of contemporary life (1) and in the health sector in particular (2). For instance, in medicine, big data are perceived as one of the major drivers of genomic medicine (3). Indeed, rapid genomic data collection on a large scale, made possible by the use of high-throughput sequencing technologies, has made the production of new medical knowledge possible. This knowledge has helped to improve disease prevention, risk prediction, individualized care, and patient involvement (4, 5). One of the conditions of such progress, however, is the need to create databases large enough to enable successful comparative analyses (6). While some initiatives seeking to share different national databases have been launched at the international level (7), the sharing of data between public institutions and private organizations remains a critical question.

Drawing on the example of databases of variants in breast and ovarian cancer predisposition BRCA 1–2 genes, we will show that genomic data is a techno-scientific democracy issue worth discussing. In this case, the recent evolution of patenting legislation has led to a shift from gene sequencing to the clinical interpretation of its results as the key activity of oncogenetics (8). Database access, which is necessary to estimate the risks associated with sequenced genetic variants, has become a critical issue, especially for private firms wishing to break into the market. In this context, the partial privatization of public databases, such as that of the French consortium that will be discussed later, is proof that there is a growing movement of public-private hybridization of these infrastructures. This shift, accentuated by the developments of high-throughput sequencing and genomic medicine, needs to be accompanied by reflection about the public health system user information contributing to the constitution of these databases.

## Patenting genes

The controversy that shook the world of genetic cancer for years is well known. Indeed, the American company Myriad Genetics filed a patent application claiming BRCA1, BRCA2, and genetic methods of diagnosing a predisposition for breast and ovarian cancer (9, 10). Thanks to the legal ownership of these genes which had been designed as biotechnologies, the start-up from Salt Lake City sought to have a global monopoly on the hereditary breast cancer market, which was expected to experience robust growth. In the face of this offensive, institutional resistance (bringing together hospitals, ministries, associations, etc.) arose in the early 2000s in Europe and then in the United States (11). This resistance has often been interpreted as paradigmatic of the opposition between an “open science,” regulated by peers respecting the law of priority, and a “proprietary science,” regulated by the market, and respecting intellectual property (12). There was thus concern that the production of public knowledge would decline because of the legal appropriation of genes by private organizations (13).

An analysis of the British case, however, helps to get a more balanced view of this dichotomy. Indeed, (14–16) has shown that patents are perceived as legal weapons by private organizations as well as by public scientific, medical, and social institutions. Moreover, actors from private and public groups cannot be radically distinguished insofar as each defines the other in a complex network of negotiated interrelationships. In line with the studies undertaken on the role of patents in management science between academic circles and the business world (17, 18), Parthasarathy calls attention to how the NHS and Myriad reached an agreement in the early 2000s, making it possible to connect the “moral order” of the former, based on the principle of equal access to healthcare for all citizens, to the freedom of consumers valued by the latter. Among the negotiated items, it appears clearly that the issue of the transfer of data from Myriad to the NHS was essential and intended to add onto the public BRCA mutation databases. Beyond the issue of monopoly over the gene sequence through the patenting of genes or methods, this example clearly shows that the ownership of data is of crucial importance to both groups. With high-throughput sequencing technology, it has become a major issue.

## Next generation sequencing

Two major developments placed the issue of the sharing of BRCA databases at the center of the debate from the 2010s. The first, naturally, was the full or partial decline in the patents claimed by Myriad Genetics around the world (19, 20). Indeed, this decline opened up the sequencing market to new private actors (GeneDx, Invitae, Pathway Genomics, Counsyl, etc.) and allowed public laboratories to carry out their activities. The second development was the progressive introduction of high-throughput DNA sequencing technology which began in the mid-2000s. The use of these “next generation” devices reinforced laboratories' analytical capacities. It is now possible to analyse within a few hours, and at the same time, several genes (panels) of several individuals, or even the complete genome of an individual at a much lower cost-100 dollars is regularly mentioned, compared to the 3 billion dollars spent in the framework of the Human Genome Project 20 years ago (21). All these developments have led stakeholders to focus on the issue of the classification of the genetic variants in BRCA genes.

A genetic variant from a sequenced individual can only acquire the status of “mutation,” i.e., the status of “pathogenic” variant, if it is clearly linked to a history of illness, either directly (in the individual or in their family) or indirectly (in a family affected by cancer and found to have the same variant). According to the current classification in genetics, the clinical significance of these variants may vary: they can be pathogenic, probably pathogenic, of unknown significance, benign, or probably benign. As (22) have pointed out, distinguishing between these categories is a major “interpretive dilemma” for geneticists. The classification of a variant in a given category depends on available data concerning the frequency of the link associating it with a specific disease. In the absence of data, the clinical significance of the variant is deemed unknown—a Variant of Unknown Significance (VUS)—until it is identified in other individuals with similar phenotypic characteristics. The importance of new DNA sequencing technologies thus lies in their ability to increase genetic databases more quickly in order to reduce the at times dramatic clinical uncertainty associated with diagnosed genetic anomalies (23). The sharing of information among geneticists, thanks to databases fed on an international scale, is a central issue^1^. This sharing of information, however, is now problematic.

## Genomic databases

For several years now, science and technology studies have been stressing that physical infrastructure plays a central role in the production of knowledge (24–27). In this area, the study of genetic databases serves as a model (28–31). Indeed, the first molecular biology databases were launched by different public institutions around the world in the early 1980s [(32): 75]. With the spread of the Internet and the Human Genome Project in the 1990s, they quickly developed as a form of support for new open “communication regimes” between scientists, likely to encourage the emergence of new knowledge (33). However, an analysis of the construction of this information infrastructure shows that the modes of data publishing remain a major source of tension between different actors.

This tension has been highlighted by Bruno Strasser, for instance, in his study on the development of the comprehensive GenBank sequence database (32). This historian of life sciences argues that tensions linked to the different conceptions of data ownership arose from the outset of the project. Participants engaged in a “moral economy of natural history,” i.e., in a “system of values that places emphasis on the exchange of scientific knowledge” inherited from the naturalists of the eighteenth century, considered that the sequences published in scientific journals should be freely accessible data. Other participants, advocates of a “moral economy of experimentation” which has garnered momentum among molecular biologists, view sequences as the products of scientific activity and as the property of their authors. According to Strasser, GenBank embodies a form of hybridization of these two value systems. It appears that those who conceived it succeeded in taking advantage of the “ambiguity” of the very notion of “data,” owing to the fact that what seems “literally given” is at the same time “the result of an organized action” (34): 248). In the context of the Human Genome Project, this ambiguity has manifested itself in the emergence of information control modes which involve a complex interplay of revelation and concealment (35). Nowadays, as seen previously, in addition to the tensions inherent in the moral economies of science, other tensions associated with the political economy of knowledge resulting from the growing role played by private firms in the production of knowledge emerged from the early 1990s (36, 37). Beyond the question of the patentability of living organisms, it is now the question of sharing that is in front of the debate, like the case of BRCA1 and BRCA2 genes clearly shows it.

## Data sharing

In the present case, i.e., the focus on BRCA1 and BRCA2 genes, there is no unique and comprehensive database of BRCA variants accessible to all professionals around the world. On the contrary, different databases developed by consortia of multinational public institutions or private organizations exist, but their access is generally limited. This is the case of the database developed by Myriad Genetics throughout the period the patents were under discussion. Although this is the largest database in the world, Myriad Genetics has exclusive access to it. This has given the company a major competitive asset in the BRCA testing market insofar as the database offers a solid basis on which to interpret results. According to genetics professionals, the main issue is not the sequencing itself. Rather, what matters most is the interpretation of the results intended to give clinical significance. This has turned out to be the most costly activity, both in terms of the recruitment of highly qualified personnel and for the development, maintenance, and access to huge databases that list the known variants of specific genes. Certain professionals estimate that there is a 1 to 10 ratio with regards to the cost of complete genome sequencing and its interpretation. In this context, ownership and the opening up of genetic variants databases emerges as a crucial issue.

From this context, the example of the future of the UMD BRCA base—*Universal Mutation Database-BRCA*—speaks volumes. Developed in the 1990s by a public consortium of French geneticists, it was considered to be one of the most important global databases until 2015. Driven by two major players in genetic testing in the United States [Quest Diagnosis and Laboratory Corporation of America (LabCorp)], the database was partially privatized in 2015. These two companies purchased the right to obtain access to data in exchange for funding the database. While the French sought to finance over the short- and medium-term an activity that had become too costly for public finances to sustain, the Americans' objective was to quickly be able to compete with Myriad Genetics by improving the quality of their analyses. The question that arises, then, is: How will this be handled over the long term? Will the French geneticists at the origin of the database still be able to access it? Will French patients still benefit from the knowledge generated thanks to the data they provided? What justifies this privatization if we consider the donations made by patients who agreed to have their data kept in this database? Similar questions had already been raised by the NHS during its negotiations with Myriad in the early 2000s, when the issue of the privatization of access to BRCA testing for British citizens arose (16). Questions revolving around access (currently and in the future) to genetic databases thus remain relevant.

## Conclusion

At a time when the opening up of public data has become common practice in the field of administration (38), the example of the genetics of breast cancer shows that data sharing is still a major issue in research (39). The question here is the extreme overlapping of public issues and private interests. In this case, there is a need to go beyond a simple comparison between the open regimes of data publication associated with academic institutions, and the closed regimes of the privatization of knowledge developed by business communities. Hybrid forms of database ownership such as those mentioned earlier, highlight the need to pay attention to the significance given to data sharing during the initial negotiations underpinning their establishment. Once these databases are filled by voluntary citizens who provide their DNA data, data sharing becomes a crucial issue in terms of technical democracy (40). Once again, however, citizens seem to be largely absent from the debate about the ownership and use of the genomic data stored in these databases. With increased power given to major programmes seeking to collect big data in genomics, it may be time to reflect on how citizens can be informed and involved in the decisions that will be made in this area.

At the very least, it seems necessary to provide people with information about the future of their genomic data: in which databases will the data be stored? For how long? Who will be able to use them? Can they be exploited for commercial purposes by private firms? As in the field of the Internet, database contributors should be able to oppose the reuse of their “data” for the benefit of private interests. The information challenge involves the very value of consent (41).

## Author contributions

The author confirms being the sole contributor of this work and has approved it for publication.

### Conflict of interest statement

The author declares that the research was conducted in the absence of any commercial or financial relationships that could be construed as a potential conflict of interest.

## Footnotes

^1^For example: Human Gene Mutation Database (HGMD) or Online Mendelian Inheritance in Man (OMIM).
